# Supplementary figures and images for: p38γ and p38δ modulate innate immune response by regulating MEF2D activation
Source: eLife. 2023 Jul 17;12:e86200. doi: 10.7554/eLife.86200 (PMC10400073; doi:10.7554/eLife.86200)

Source Data: Figure 1A

A

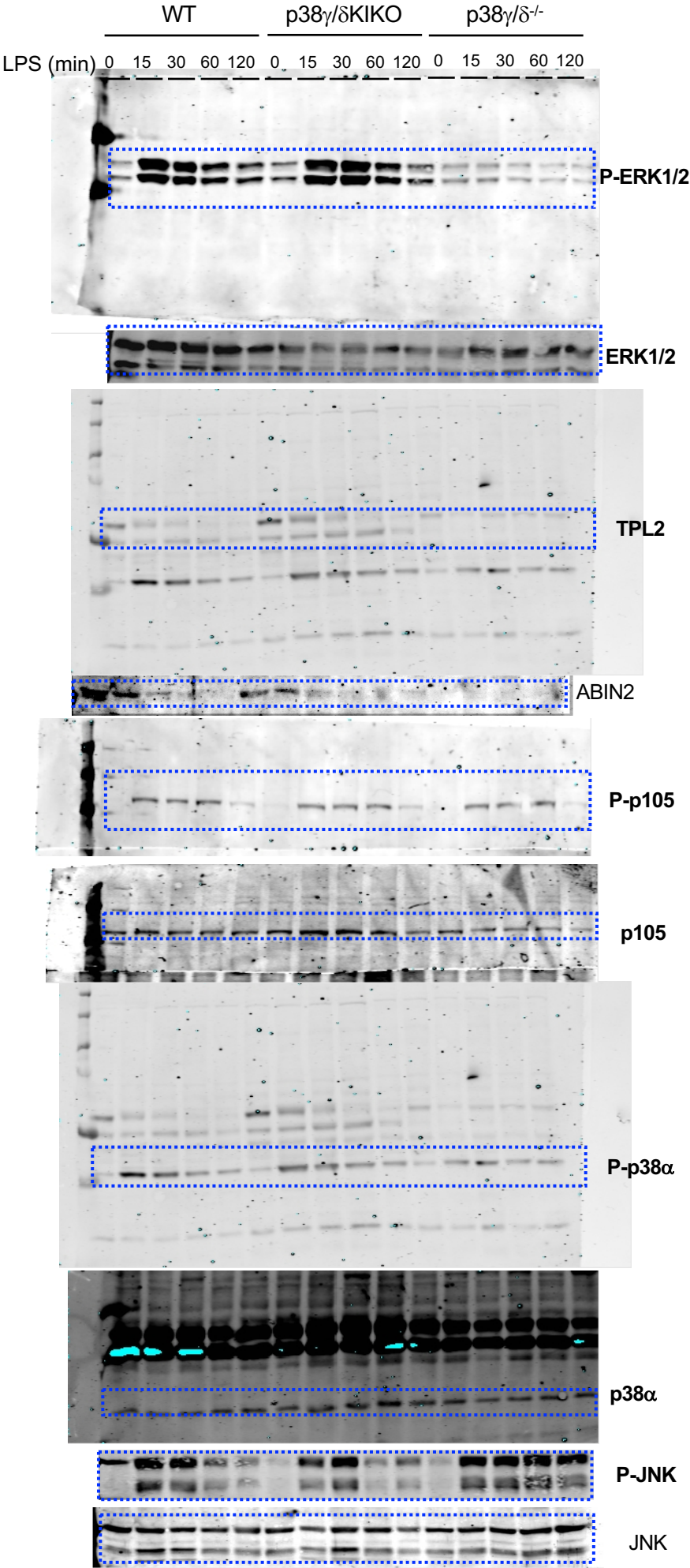

Supplement: Figure 1—source data 1. [file elife-86200-fig1-data1.zip › Figure 1-source data 1/Figure 1-source data 1.pdf]

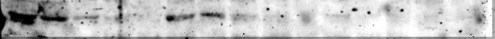

Supplement: Figure 1—source data 1. [file elife-86200-fig1-data1.zip › Figure 1-source data 1/ABIN2.jpg]

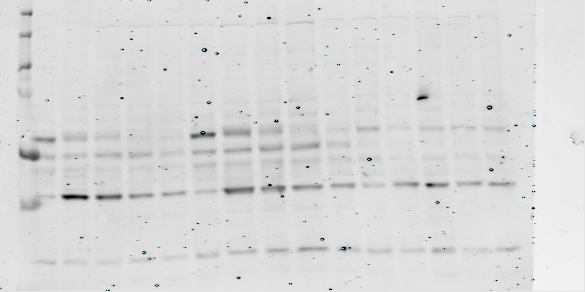

Supplement: Figure 1—source data 1. [file elife-86200-fig1-data1.zip › Figure 1-source data 1/Tpl2.jpg]

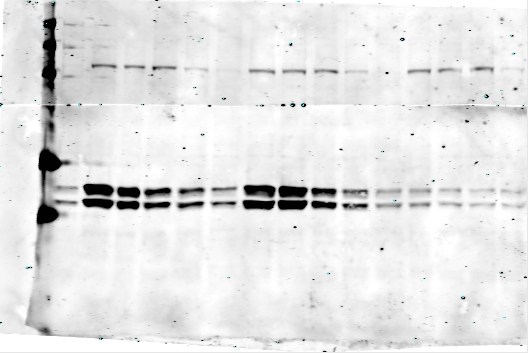

Supplement: Figure 1—source data 1. [file elife-86200-fig1-data1.zip › Figure 1-source data 1/Pp105 .jpg]

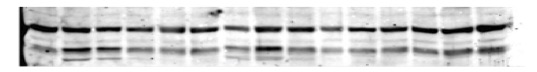

Supplement: Figure 1—source data 1. [file elife-86200-fig1-data1.zip › Figure 1-source data 1/JNK.jpg]

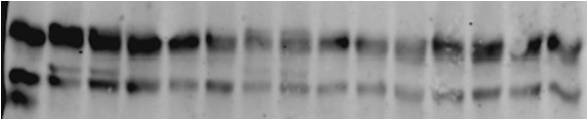

Supplement: Figure 1—source data 1. [file elife-86200-fig1-data1.zip › Figure 1-source data 1/ERK12.jpg]

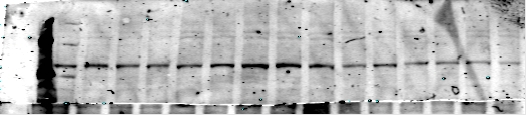

Supplement: Figure 1—source data 1. [file elife-86200-fig1-data1.zip › Figure 1-source data 1/P105.jpeg]

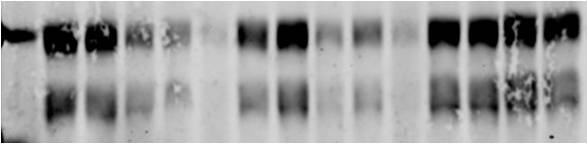

Supplement: Figure 1—source data 1. [file elife-86200-fig1-data1.zip › Figure 1-source data 1/PJNK12.jpg]

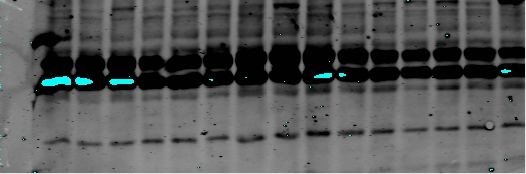

Supplement: Figure 1—source data 1. [file elife-86200-fig1-data1.zip › Figure 1-source data 1/p38a.jpg]

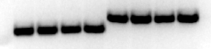

Supplement: Figure 1—figure supplement 1—source data 1. [file elife-86200-fig1-figsupp1-data1.zip › Figure 1-figure supplement 1-source data 1/PCR 3KI copy.tif]

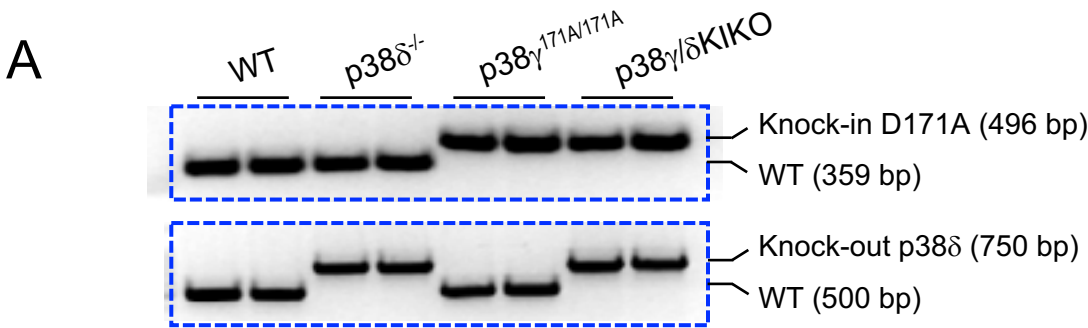

Supplement: Figure 1—figure supplement 1—source data 1. [file elife-86200-fig1-figsupp1-data1.zip › Figure 1-figure supplement 1-source data 1/Figure 1- Figure Supplement 1A.pdf]

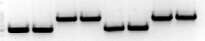

Supplement: Figure 1—figure supplement 1—source data 1. [file elife-86200-fig1-figsupp1-data1.zip › Figure 1-figure supplement 1-source data 1/2014-06-20 16hr 51min sk4 copy.tif]

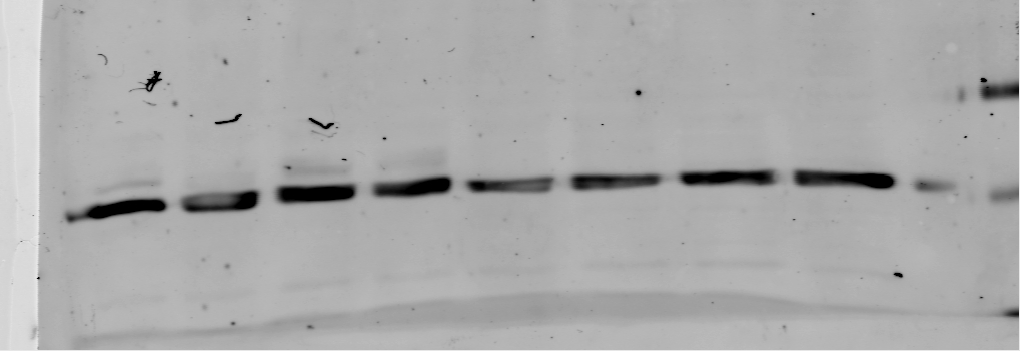

Supplement: Figure 1—figure supplement 1—source data 2. [file elife-86200-fig1-figsupp1-data2.zip › Figure 1-figure supplement 1-source data 2/p38a.jpeg]

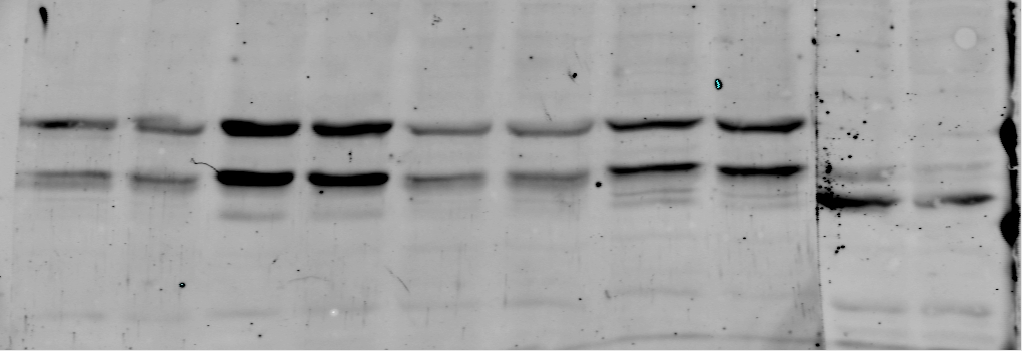

Supplement: Figure 1—figure supplement 1—source data 2. [file elife-86200-fig1-figsupp1-data2.zip › Figure 1-figure supplement 1-source data 2/ PJNK .jpeg]

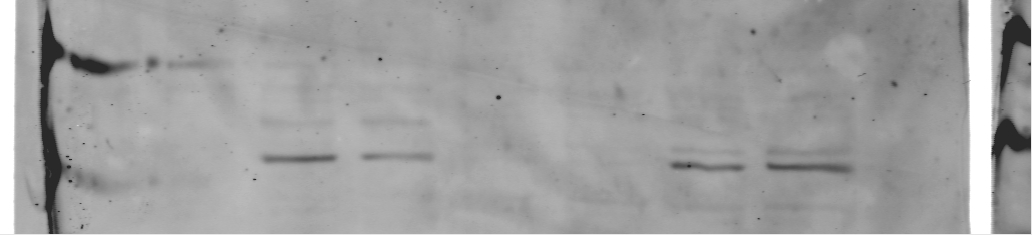

Supplement: Figure 1—figure supplement 1—source data 2. [file elife-86200-fig1-figsupp1-data2.zip › Figure 1-figure supplement 1-source data 2/Pp38a.jpeg]

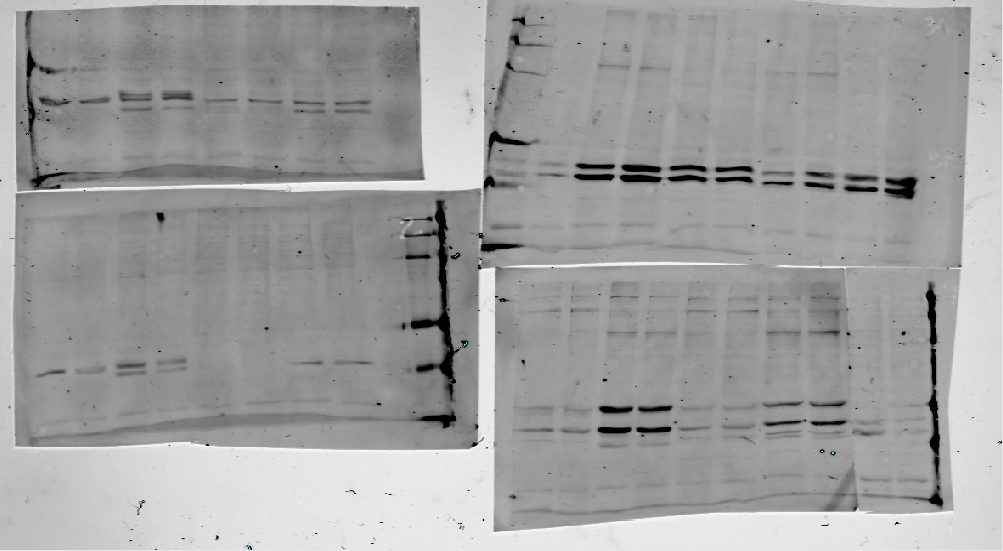

Supplement: Figure 1—figure supplement 1—source data 2. [file elife-86200-fig1-figsupp1-data2.zip › Figure 1-figure supplement 1-source data 2/p38g_p38d copy.jpeg]

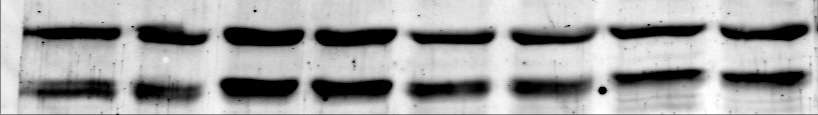

Supplement: Figure 1—figure supplement 1—source data 2. [file elife-86200-fig1-figsupp1-data2.zip › Figure 1-figure supplement 1-source data 2/JNK 6.0_B copy.jpeg]

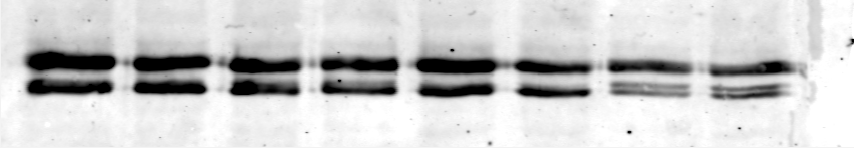

Supplement: Figure 1—figure supplement 1—source data 2. [file elife-86200-fig1-figsupp1-data2.zip › Figure 1-figure supplement 1-source data 2/2014-06-24 MEFs ERK1.2 Q 5.0_1 copy.tiff]

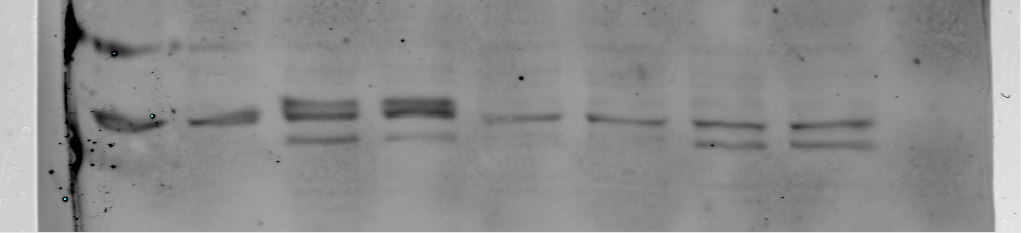

Supplement: Figure 1—figure supplement 1—source data 2. [file elife-86200-fig1-figsupp1-data2.zip › Figure 1-figure supplement 1-source data 2/MEFS 27.03.14 p38g 5.0 Q_B_1 copy.jpg]

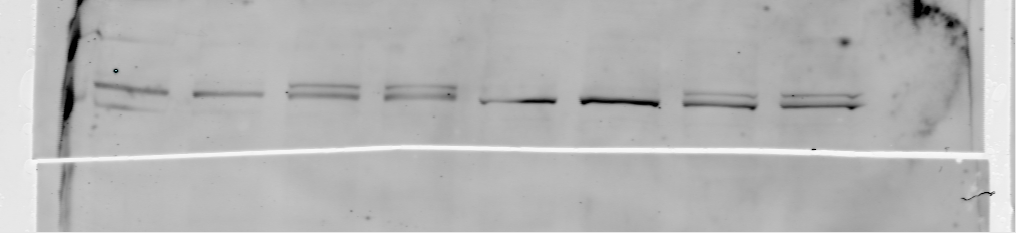

Supplement: Figure 1—figure supplement 1—source data 2. [file elife-86200-fig1-figsupp1-data2.zip › Figure 1-figure supplement 1-source data 2/ERK5 5.0_B copy.jpeg]

B

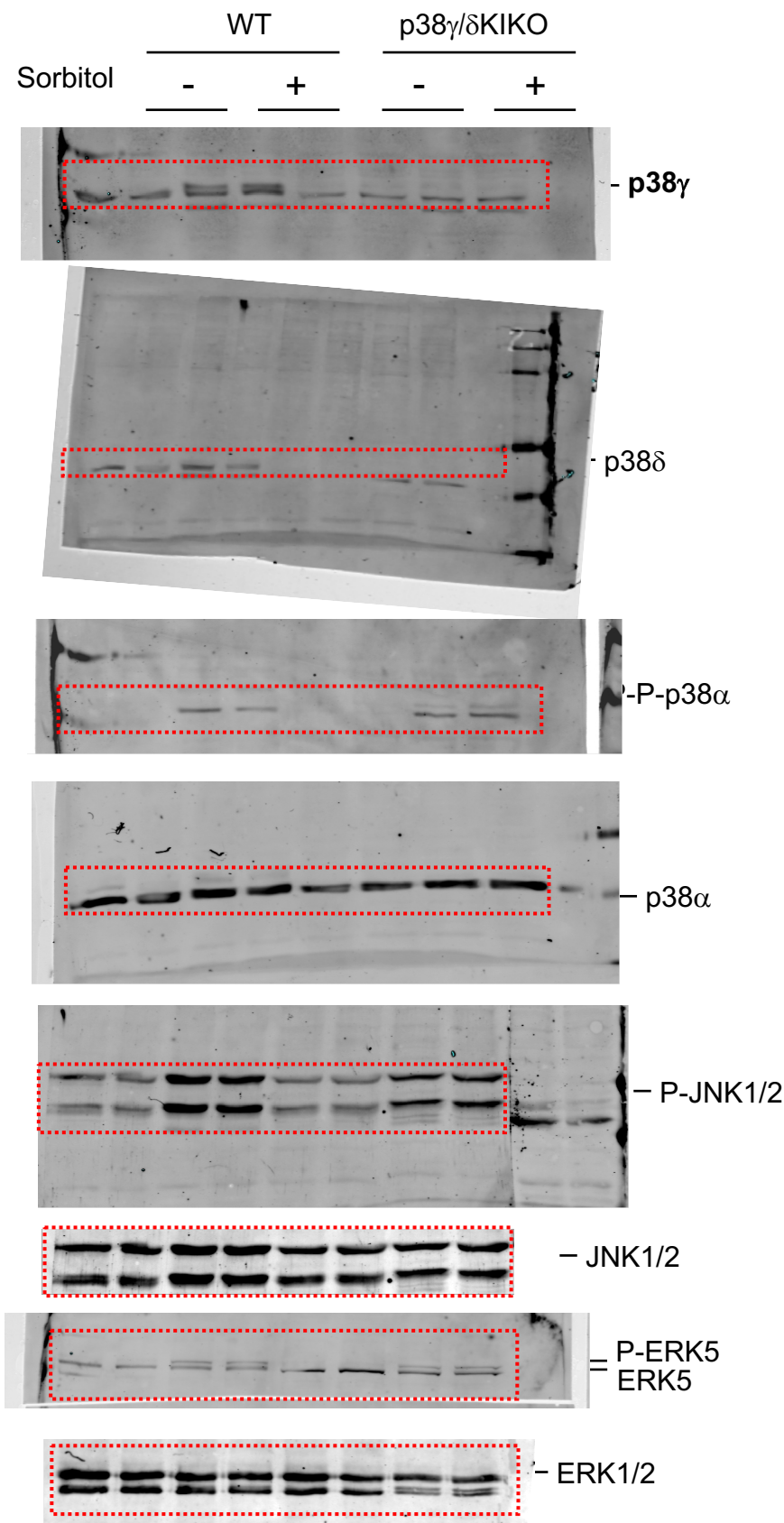

Supplement: Figure 1—figure supplement 1—source data 2. [file elife-86200-fig1-figsupp1-data2.zip › Figure 1-figure supplement 1-source data 2/Figure 1-figure supplement 1-source data 2.pdf]

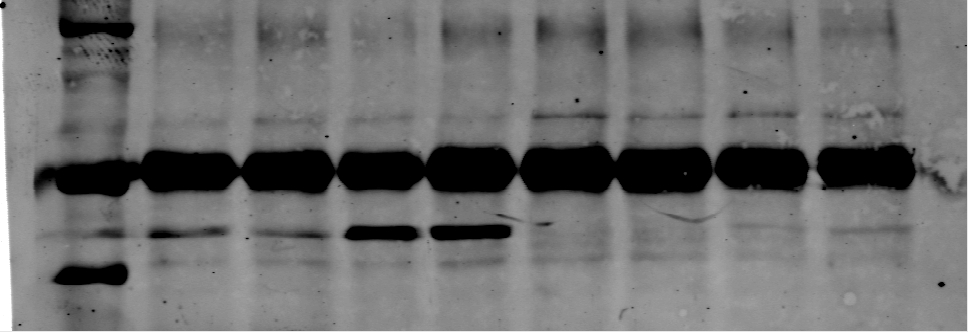

Supplement: Figure 1—figure supplement 1—source data 3. [file elife-86200-fig1-figsupp1-data3.zip › Figure 1-figure supplement 1-source data 3/11.04.14 MEFs IP SK3 5.5Q_B copy.jpg]

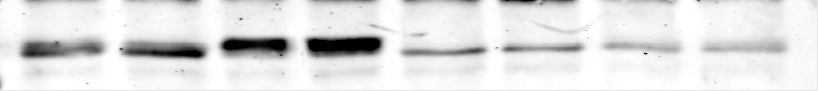

Supplement: Figure 1—figure supplement 1—source data 3. [file elife-86200-fig1-figsupp1-data3.zip › Figure 1-figure supplement 1-source data 3/11.04.14 MEFs IP SK3 Q 5.0_B copy.jpg]

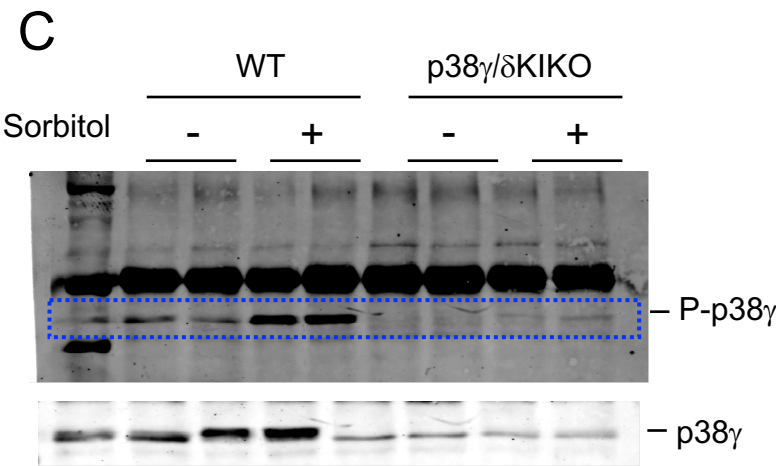

Supplement: Figure 1—figure supplement 1—source data 3. [file elife-86200-fig1-figsupp1-data3.zip › Figure 1-figure supplement 1-source data 3/Figure 1-figure supplement 1-source data 3.pdf]

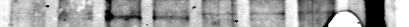

Supplement: Figure 1—figure supplement 1—source data 4. [file elife-86200-fig1-figsupp1-data4.zip › Figure 1-figure supplement 1-source data 4/IP_SAP97SER158_30.01.14_B copy.jpg]

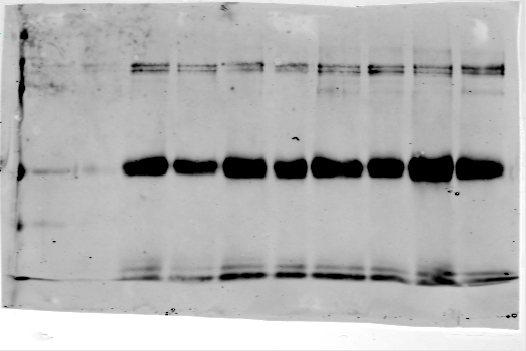

Supplement: Figure 1—figure supplement 1—source data 4. [file elife-86200-fig1-figsupp1-data4.zip › Figure 1-figure supplement 1-source data 4/18.12.13 MEFs P SAP97 5.0_BN copy.jpg]

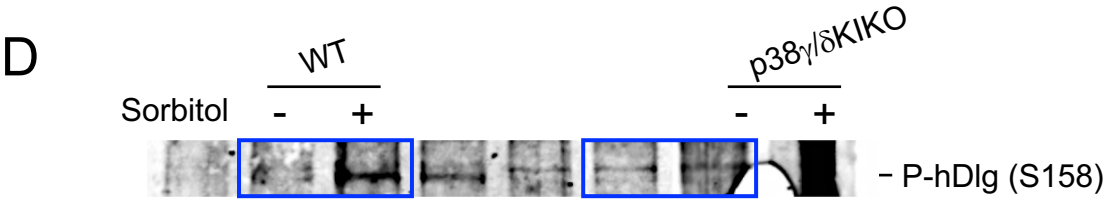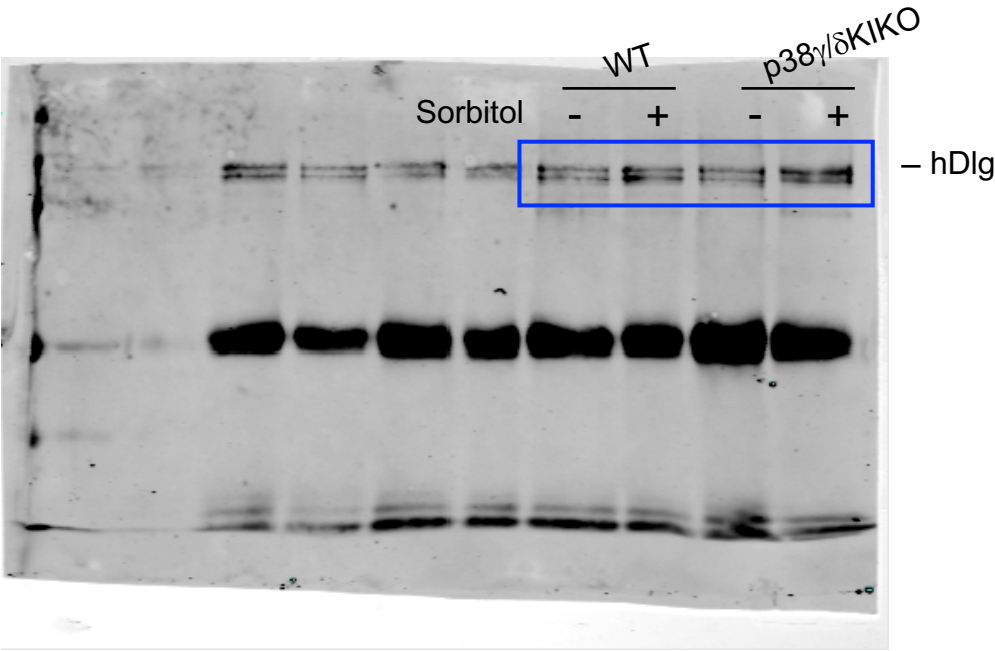

Supplement: Figure 1—figure supplement 1—source data 4. [file elife-86200-fig1-figsupp1-data4.zip › Figure 1-figure supplement 1-source data 4/Figure 1-figure supplement 1-source data 4.pdf]

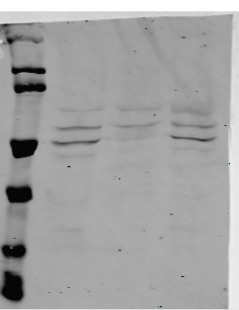

Supplement: Figure 1—figure supplement 1—source data 5. [file elife-86200-fig1-figsupp1-data5.zip › Figure 1-figure supplement 1-source data 5/TPL2.jpg]

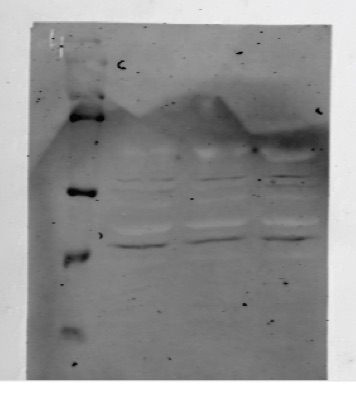

Supplement: Figure 1—figure supplement 1—source data 5. [file elife-86200-fig1-figsupp1-data5.zip › Figure 1-figure supplement 1-source data 5/p38.jpg]

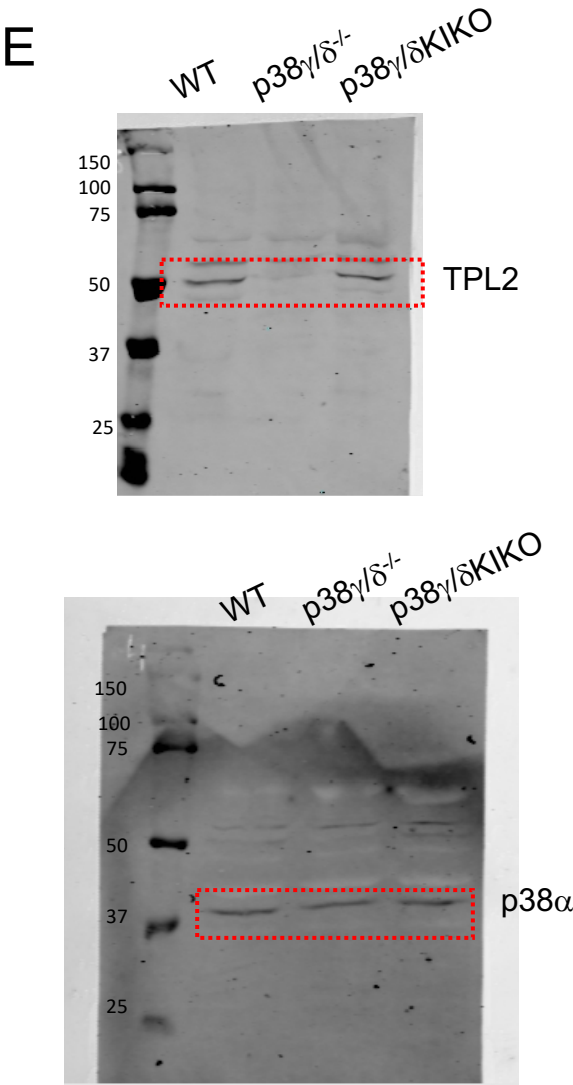

Supplement: Figure 1—figure supplement 1—source data 5. [file elife-86200-fig1-figsupp1-data5.zip › Figure 1-figure supplement 1-source data 5/Figure 1-figure supplement 1-source data 5.pdf]

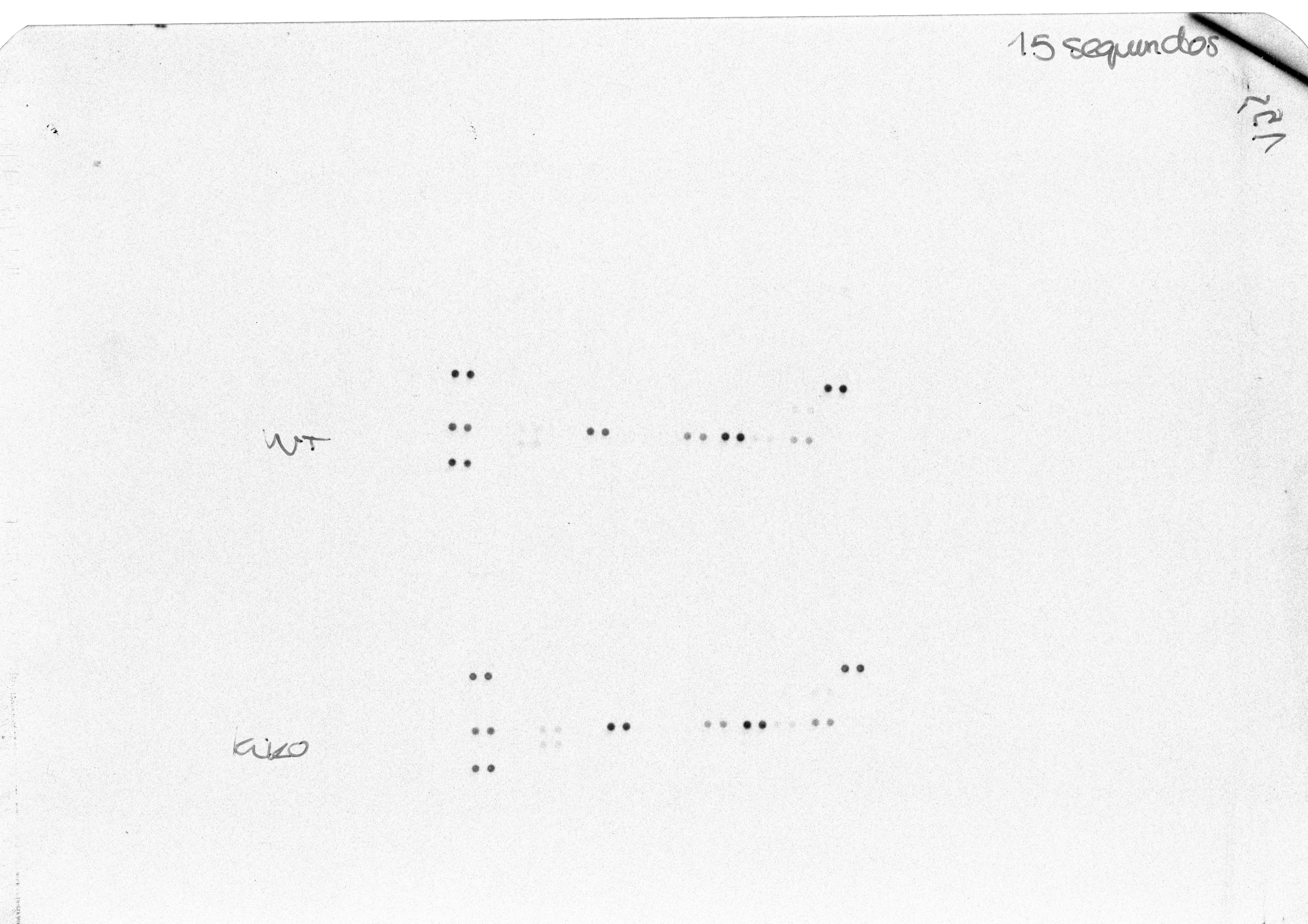

Supplement: Figure 3—figure supplement 1—source data 2. [file elife-86200-fig3-figsupp1-data2.zip › Figure 3-figure supplement 3-source data 2/Wt Kiko 15seg048 copy.tif]

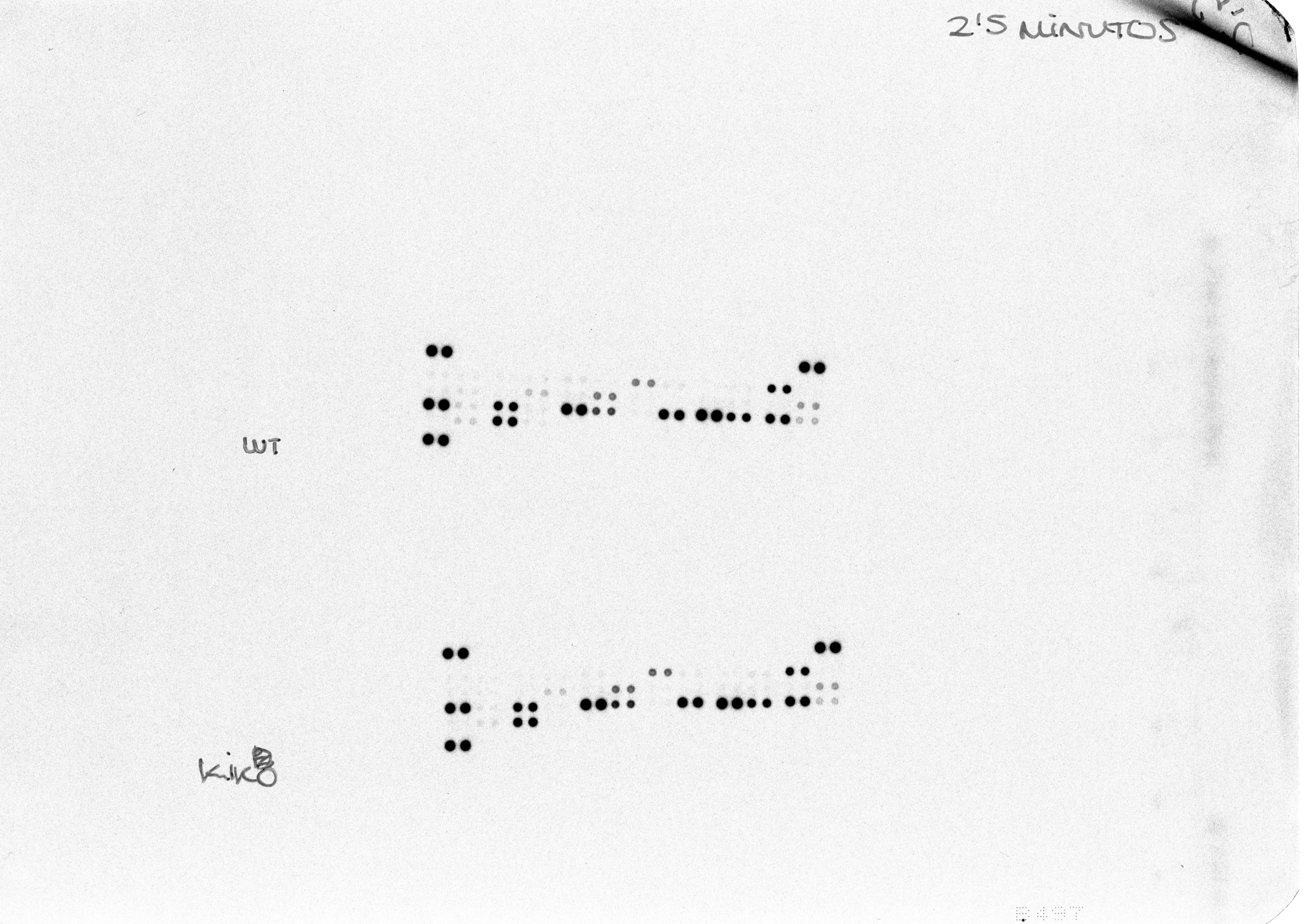

Supplement: Figure 3—figure supplement 1—source data 2. [file elife-86200-fig3-figsupp1-data2.zip › Figure 3-figure supplement 3-source data 2/Wt Kiko 2,5 min044 copy.tif]

C

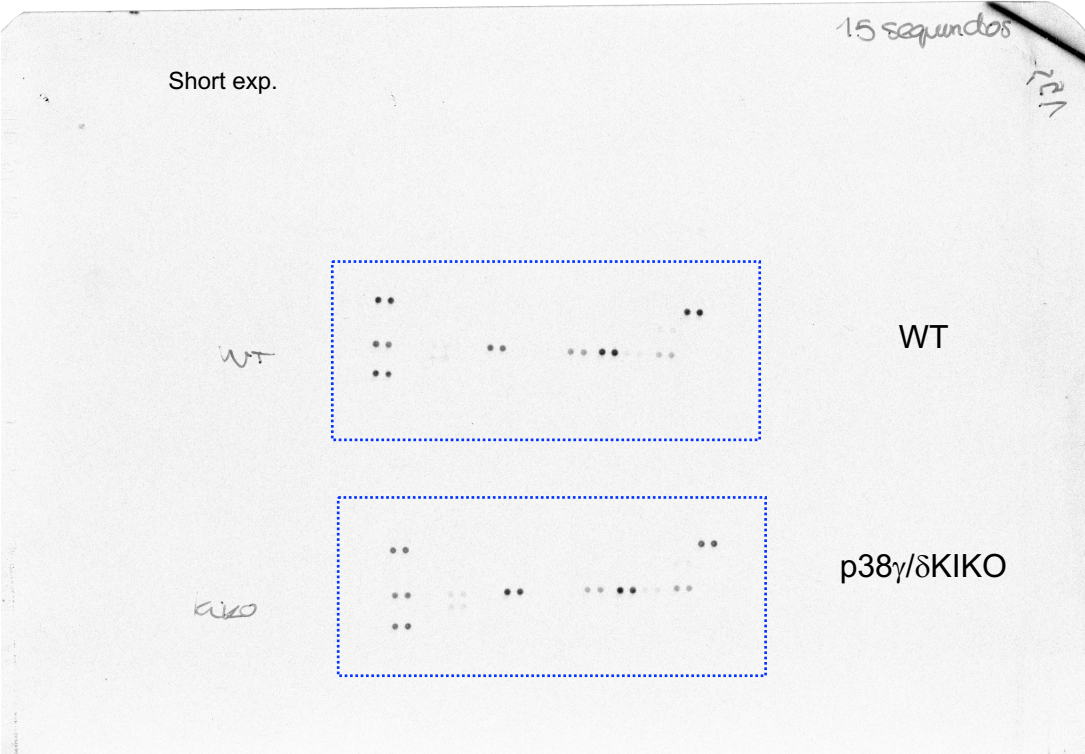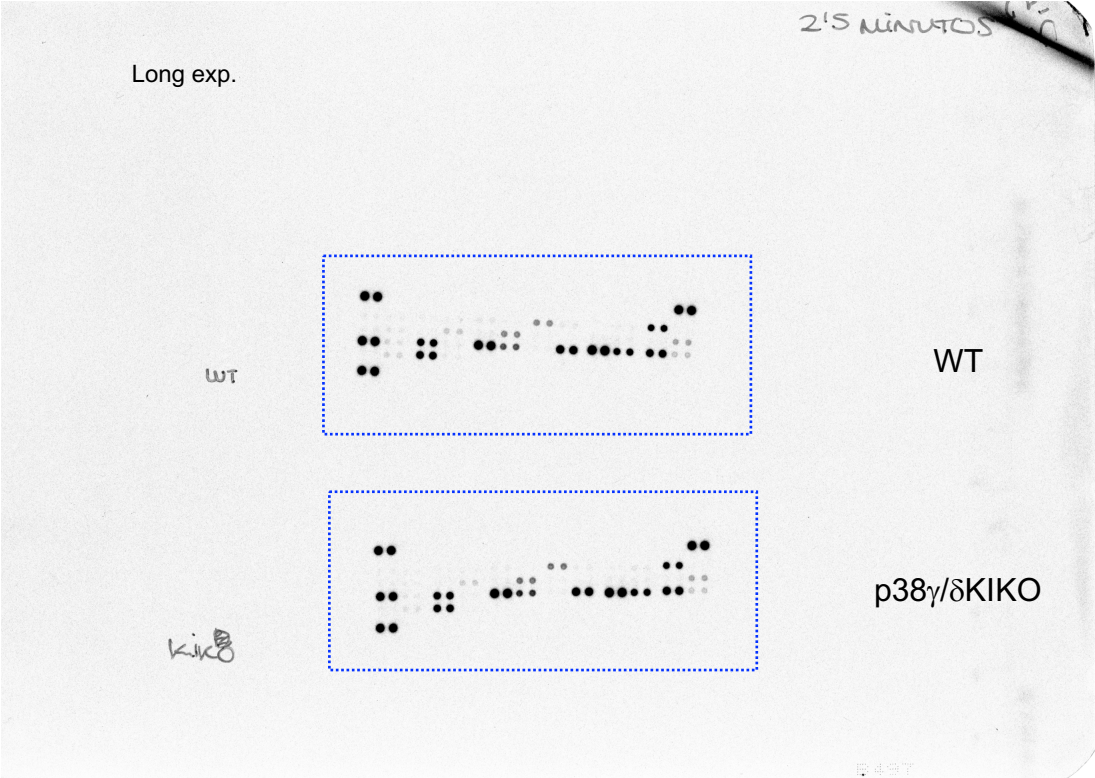

Supplement: Figure 3—figure supplement 1—source data 2. [file elife-86200-fig3-figsupp1-data2.zip › Figure 3-figure supplement 3-source data 2/Figure 3-figure supplement 3-source data 2.pdf]

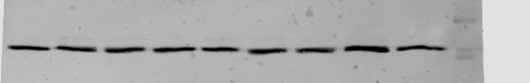

Supplement: Figure 4—figure supplement 1—source data 1. [file elife-86200-fig4-figsupp1-data1.zip › Figure 4-figure supplement 4-source data 1/AR100221mfgsperitLPSwtKIKODKp38a para Acuenda.jpg]

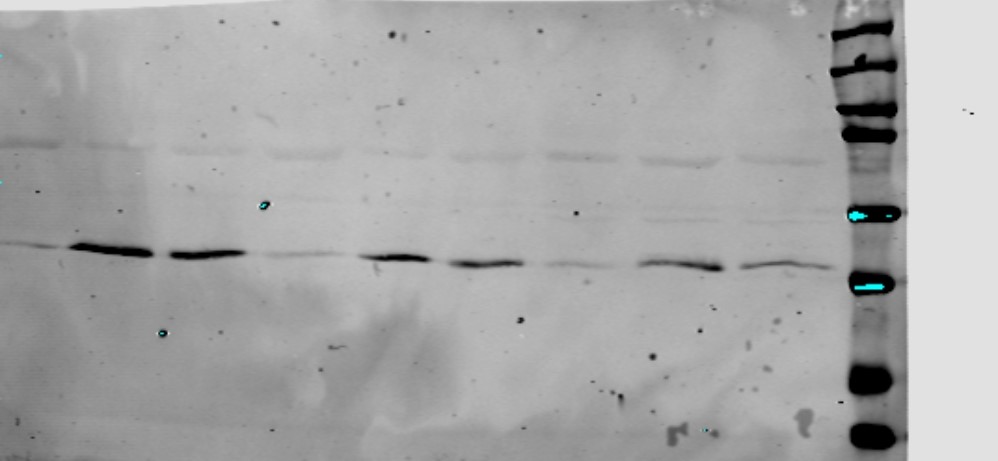

Supplement: Figure 4—figure supplement 1—source data 1. [file elife-86200-fig4-figsupp1-data1.zip › Figure 4-figure supplement 4-source data 1/WB050221para AcuendaPp38.jpg]

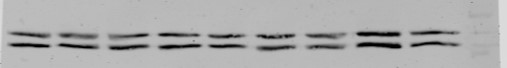

Supplement: Figure 4—figure supplement 1—source data 1. [file elife-86200-fig4-figsupp1-data1.zip › Figure 4-figure supplement 4-source data 1/AR110221mfgsperitLPSwtKIKODKerk12 para Acuenda.jpg]

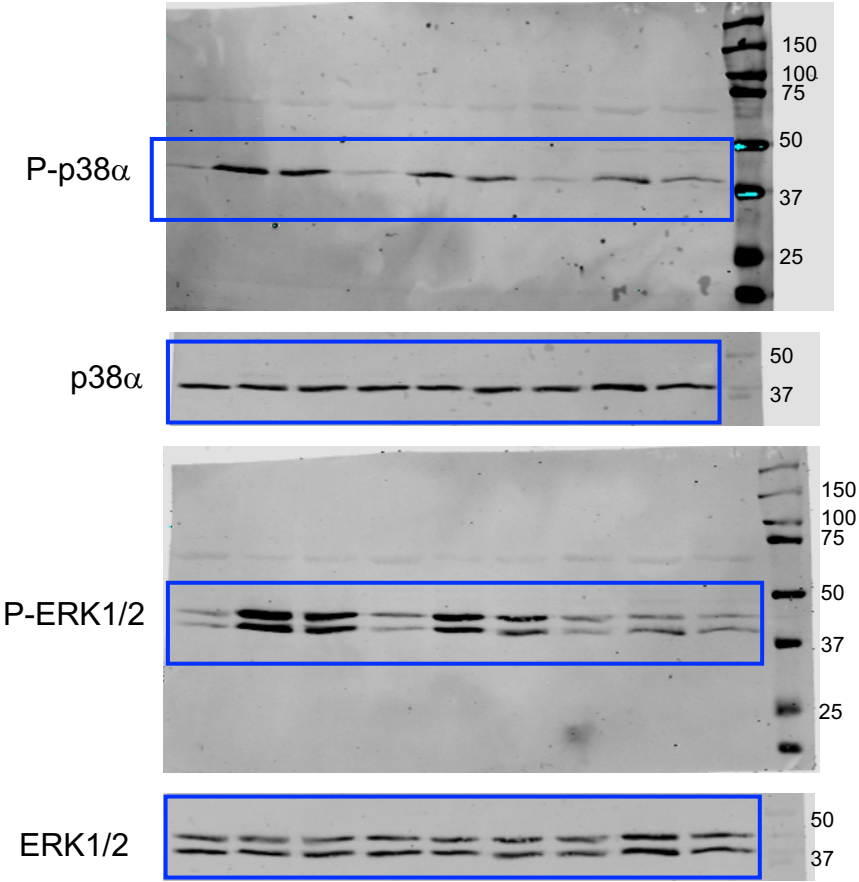

Supplement: Figure 4—figure supplement 1—source data 1. [file elife-86200-fig4-figsupp1-data1.zip › Figure 4-figure supplement 4-source data 1/Figure 4-figure supplement 4-source data 1.pdf]

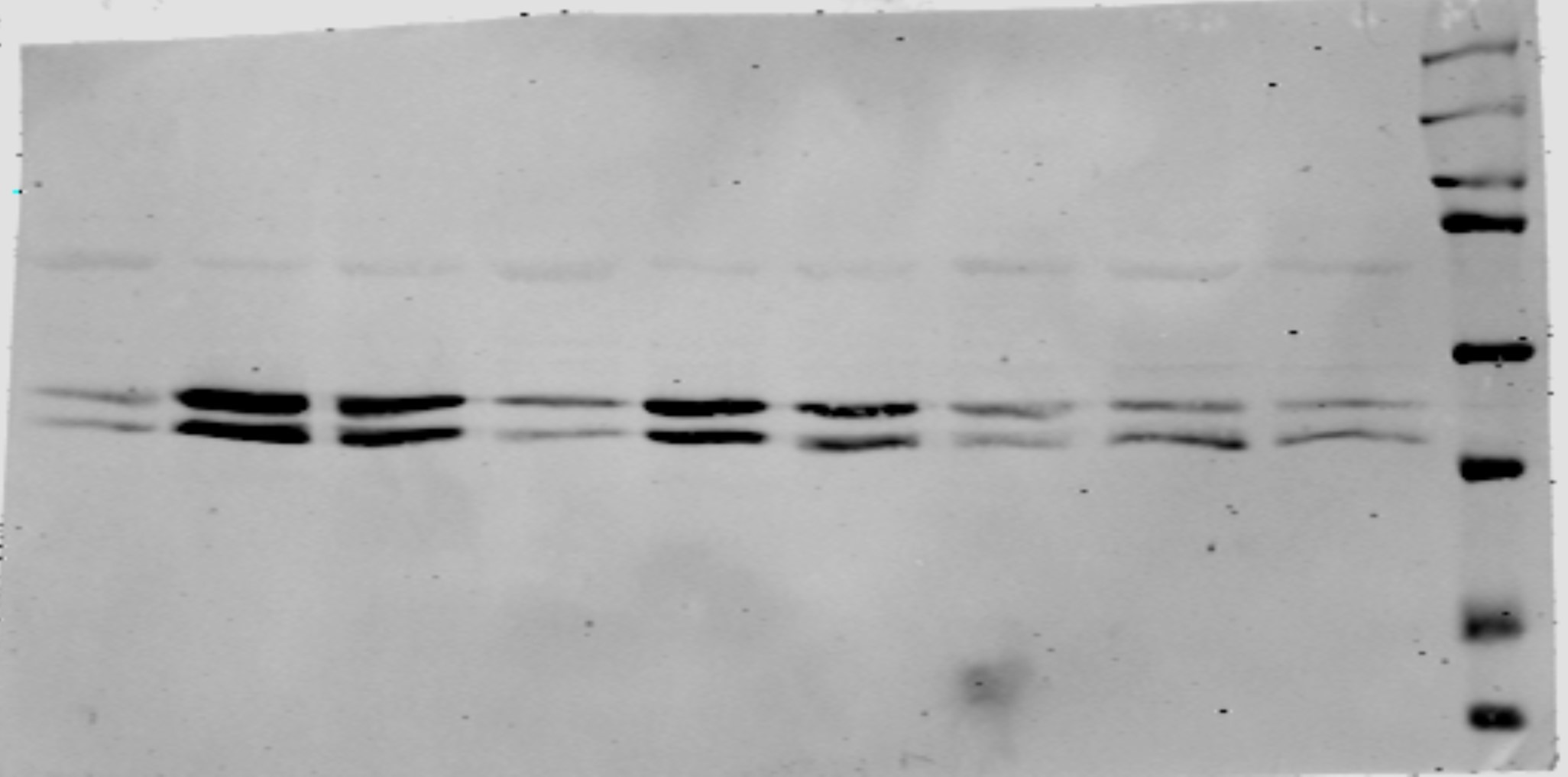

Supplement: Figure 4—figure supplement 1—source data 1. [file elife-86200-fig4-figsupp1-data1.zip › Figure 4-figure supplement 4-source data 1/WB080212 para AcuendaPerk.jpg]

## Slide 1
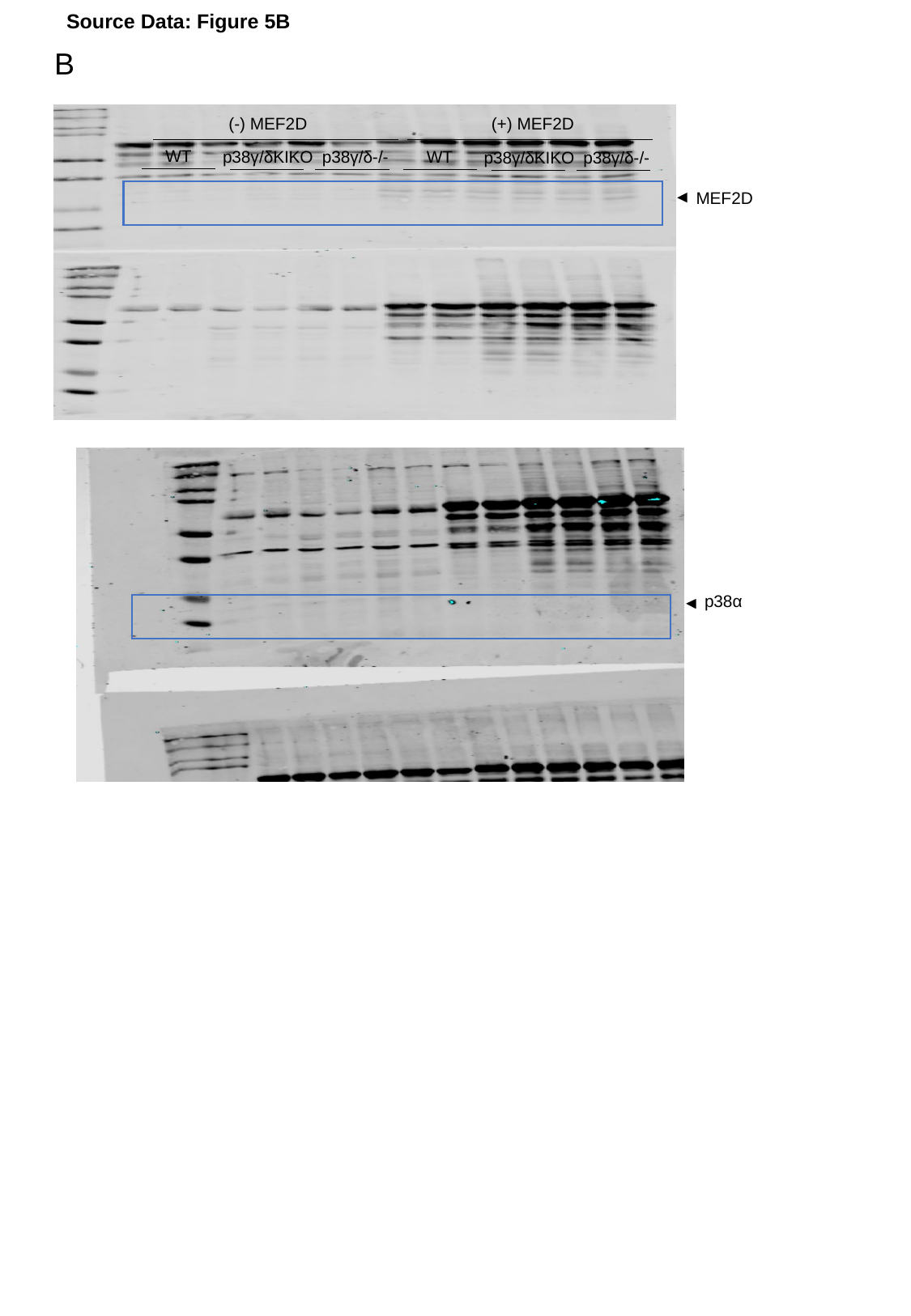

Source Data: Figure 5B
B
(-) MEF2D
(+) MEF2D
WT
WT
p38γ/δKIKO
p38γ/δ-/-
p38γ/δKIKO
p38γ/δ-/-
100
75
MEF2D
p38α
37

Supplement: Figure 5—source data 1. [file elife-86200-fig5-data1.zip › Source data Fig 5B.pptx]

## Slide 1
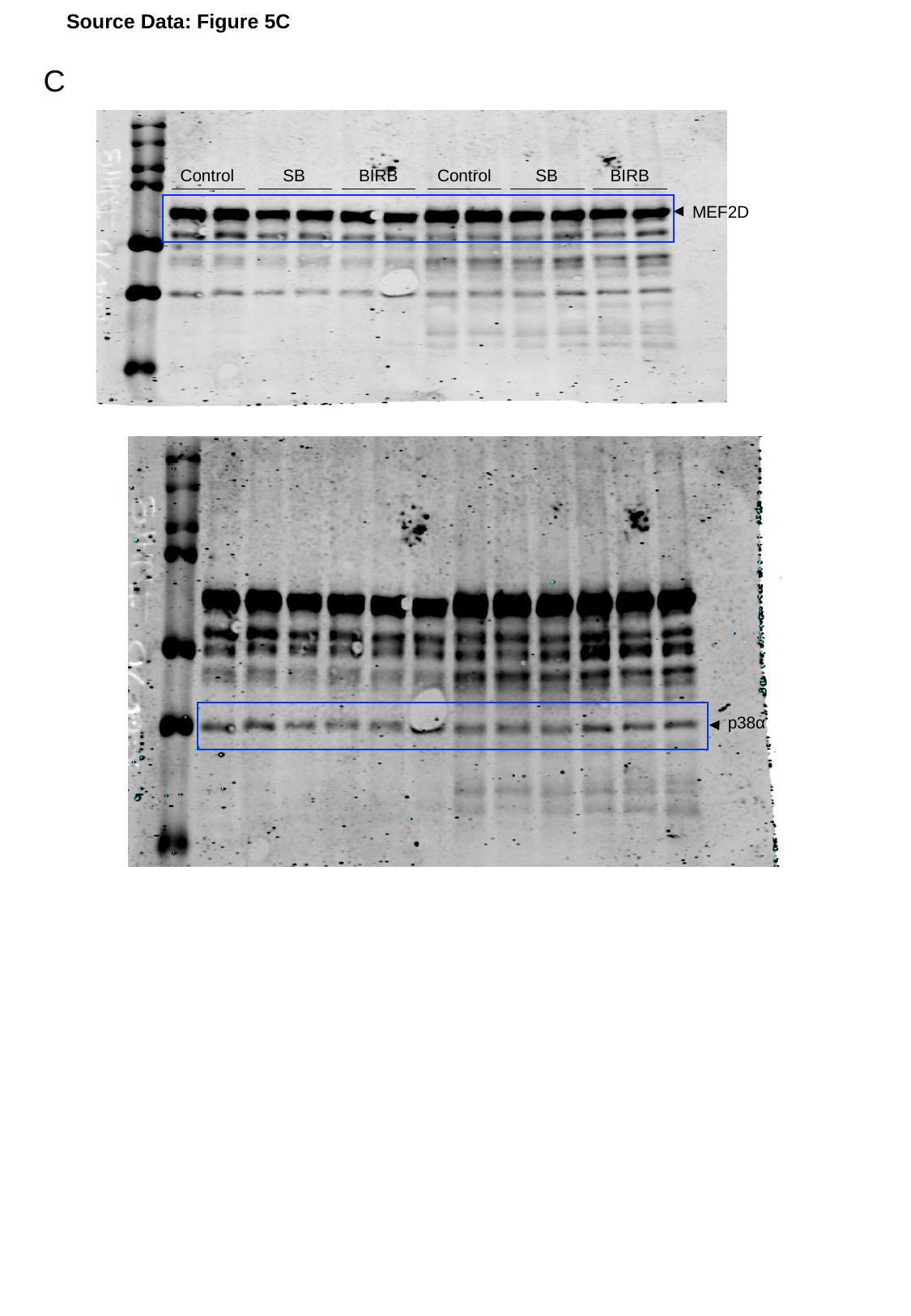

Source Data: Figure 5C
C
Control
SB
BIRB
Control
SB
BIRB
100
75
MEF2D
p38α
37

Supplement: Figure 5—source data 2. [file elife-86200-fig5-data2.zip › Source data Fig 5C.pptx]

## Slide 1
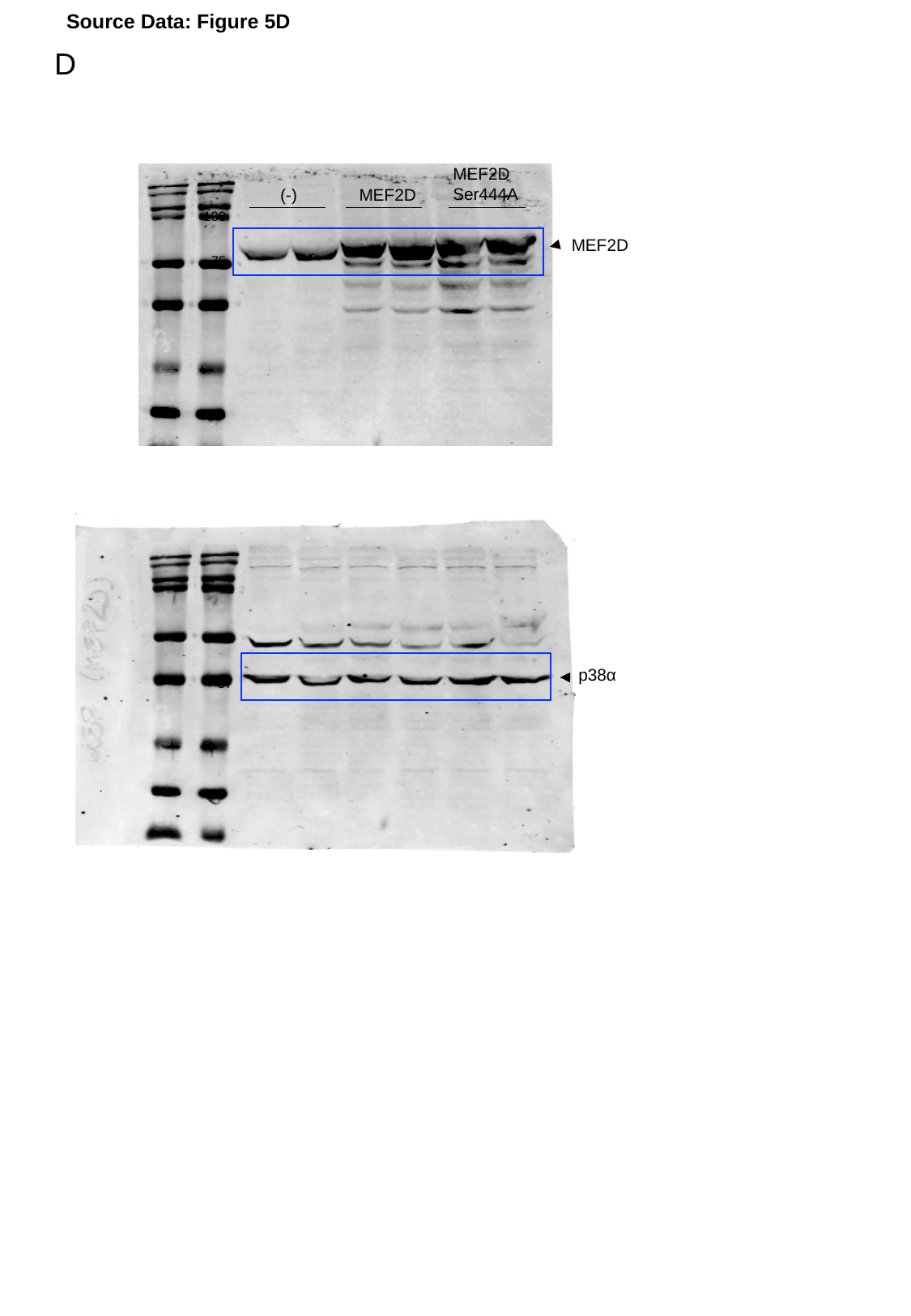

Source Data: Figure 5D
D
MEF2D
Ser444A
(-)
MEF2D
100
MEF2D
75
p38α
37

Supplement: Figure 5—source data 3. [file elife-86200-fig5-data3.zip › Source data Fig 5D.pptx]
